# The *lin-4* Gene Controls Fat Accumulation and Longevity in *Caenorhabditis elegans*

**DOI:** 10.3390/ijms11124814

**Published:** 2010-11-25

**Authors:** Chun Zhu, Chen-Bo Ji, Chun-Mei Zhang, Chun-Lin Gao, Jin-Gai Zhu, Da-Ni Qin, Chun-Zhao Kou, Guan-Zhong Zhu, Chun-Mei Shi, Xi-Rong Guo

**Affiliations:** 1 Department of Pediatrics, Nanjing Maternal and Child Health Hospital of Nanjing Medical University, No.123 Tianfei Road, 210004 Nanjing, China; E-Mails: zhifangxibao@163.com(C.Z.); zhangcm79@163.com (C.-M.Z.); 2 Institute of Pediatrics, Nanjing Medical University, No.140 Hanzhong Road, 210029 Nanjing, China; E-Mails: Jiliu1036@163.com (C.-B.J.); shuangmu34@sina.com (C.-L.G.); zhujingai1983@163.com (J.-G.Z.); qindani@yahoo.com.cn (D.-N.Q.); kou19850112@163.com (C.-Z.K); aerozgz@yahoo.com.cn (G.-Z.Z.); shichunmei_87@163.com (C.-M.S.)

**Keywords:** lin-4, Caenorhabditis elegans, fat accumulation, life span, locomotion

## Abstract

Previous studies have determined that *lin-4*, which was the first miRNA to be discovered, controls the timing of cell fate determination and life span in *Caenorhabditis elegans*. However, the mechanism of *lin-4* involvement in these processes remains poorly understood. Fat storage is an essential aspect of the life cycle of organisms, and the function of *lin-4* in fat accumulation is not clear. In this study, we showed that the fat content is reduced remarkably in *C. elegans lin-4* mutants. Quantitative RT-PCR analysis revealed a considerable decrease in the levels of SBP-1 and OGA-1 mRNA in *lin-4* mutants. We also showed that *lin-4* mutants have a significantly shorter life span than wild-type worms. DCF assay experiments showed that the reactive oxygen species (ROS) levels increased and mitochondrial DNA (mtDNA) copy number decreased in loss-of-function *lin-4* mutants. These mutants also showed attenuation of locomotion. Taken together, our findings suggest that *lin-4* may play an important role in regulating fat accumulation and locomotion and that *lin-4* may control the life span of *C. elegans* by mediating ROS production.

## 1. Introduction

MicroRNAs (miRNAs) are 18–25-nucleotide-long single-stranded RNAs that are involved in the post-transcriptional regulation of gene expression by base pairing with complementary sequences in the 3′ untranslated regions (3′ UTRs) of protein-coding transcripts. This interaction leads to the inhibition of translation [1,2]. Although the biological functions of most miRNAs are unknown, miRNAs are predicted to regulate up to 30% of the genes within the human genome [3]. Emerging evidence also suggests that miRNAs play important regulatory roles in a variety of biological processes, including adipocyte differentiation, metabolic integration, insulin resistance, and appetite regulation [4]. Although the *Caenorhabditis elegans* genome encodes over 100 miRNAs, the functions of only five have been elucidated, and less than 15 valid *C. elegans* target genes are known [5]. As the first miRNA discovered, *lin-4* acts as a developmental switch that controls the timing of cell fate determination during larval transitions in *C. elegans* and regulates the life span of the adults [6,7]. However, our current knowledge of the connection between *lin-4*, life span, and locomotion in *C. elegans* is very limited.

The increasing prevalence of obesity has significant implications for the health of humans because obesity is associated with serious co-morbidities, including type 2 diabetes mellitus, hyperlipidemia, and hypertension [8]. Obesity results from a prolonged imbalance in energy homeostasis between caloric intake and expenditure. Animal models have provided an understanding of the basic parameters that regulate the components of energy balance [9]. The use of invertebrates such as *C. elegans*, which is an excellent model for many biological processes, may be a more efficient method of screening research materials and a powerful system for analyzing the mechanisms of fat storage. Moreover, many components regulating human metabolism, including the biochemical pathways of fats, carbohydrates, and protein synthesis, are conserved in the nematode *C. elegans* [10,11]. However, the relationship between *lin-4* and fat accumulation is unknown.

In this study, we compared fat accumulation, reactive oxygen species (ROS) levels, mitochondrial DNA (mtDNA) copy number, life span, and locomotion in *lin-4* mutants with those in wild-type *C. elegans*. We also tested whether feeding glucose and fatty acids affects fat storage in *C. elegans*. In addition, we used the real-time quantitative reverse transcriptase-polymerase chain reaction (RT-PCR) to determine the mRNA expression of SBP-1 and OGA-1, which are key genes in regulating and controlling the amount and composition of fat [12,13].

## 2. Results and Discussion

### 2.1. The Fat Content Is Remarkably Reduced in *lin-4* Mutants

**Figure 1.**
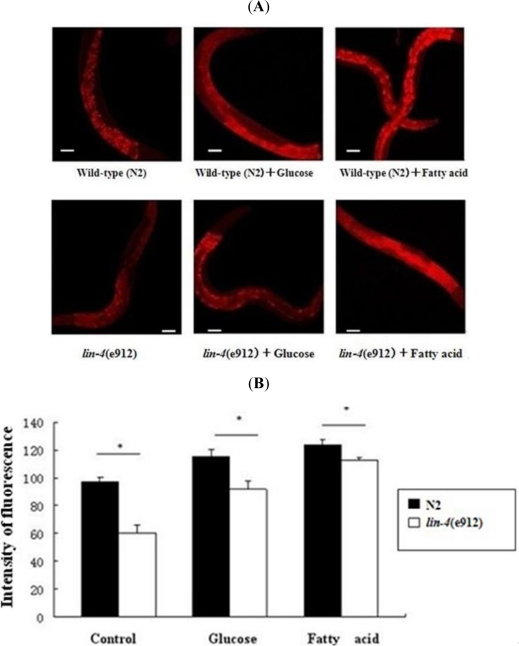
*lin-4* regulates fat storage. (**A**) Nile red staining of wild-type (N2) and *lin-4* mutant (e912) worms fed a normal diet of *E. coli* OP50 bacteria (left panel) or OP50 bacteria supplemented with glucose (middle) or fatty acid (right). Scale bar: 50 μm. *n* = 40 (*n*: number); (**B**) Quantification of the Nile red staining of fat in wild-type (N2) and *lin-4* mutant (e912) worms. *n* = 40. Error bars indicate standard error. * p < 0.05.

**Figure 2.**
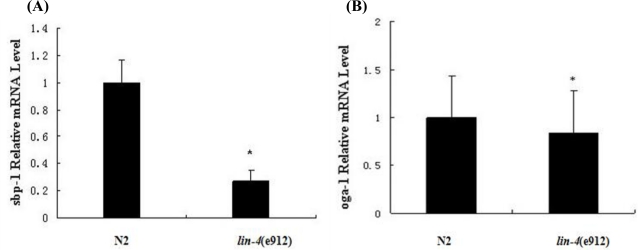
The expression of central regulation factors involved in fat storage control in nematodes. (**A**) Quantitative real-time PCR of sbp-1 in wild-type (N2) and *lin-4* mutant (e912) worms. *n* = 50 (*n*: number); (**B**) Quantitative real-time PCR of *oga-1* in wild-type (N2) and *lin-4* mutant (e912) worms. *n* = 40. Error bars indicate standard error. * p < 0.05.

As shown in Figure 1, when worms were fed a normal diet of *E. coli* OP50 bacteria, animals with a loss-of-function mutation in *lin-4* showed fat content that was significantly lower than that in wild-type animals. After adding glucose and fatty acid to culture dishes containing *C. elegans*, we found an increase in fluorescence in Nile red staining, thereby indicating an increase in intracellular fat. However, fat levels were also reduced considerably in *lin-4* mutants compared to wild-type animals. To determine whether the key genes in the metabolic pathways for fatty acid synthesis in nematodes are involved in regulating fat accumulation, we measured mRNA levels of SBP-1 and OGA-1 by using quantitative RT-PCR analysis. We found that both SBP-1 and OGA-1 mRNA levels were reduced in *lin-4* mutants, especially SBP-1(Figure 2).

### 2.2. ROS Accumulation and Decreased Copy Number of mtDNA in *lin-4* Mutants

**Figure 3.**
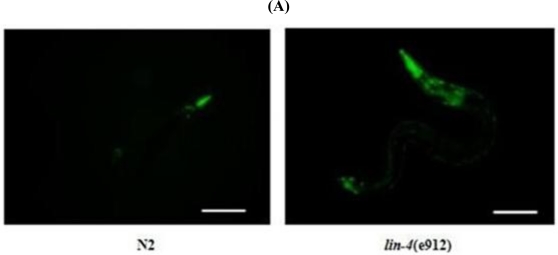

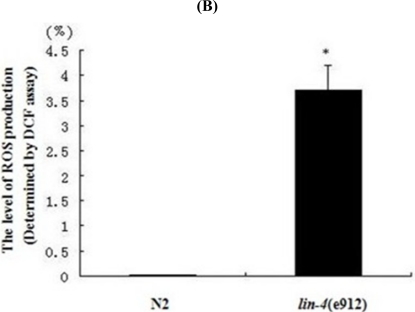
The effect of *lin-4* on ROS level. (**A**) Intracellular ROS in *C. elegans* were measured using 2,7-dichlorofluorescein diacetate (DCF-DA; Molecular Probes). Sixty animals from each group were analyzed (*n* = 60). Scale bar: 100 μm; (**B**) results are expressed as mean ± SD of relative fluorescence units (RFU). * p < 0.05.

**Figure 4.**
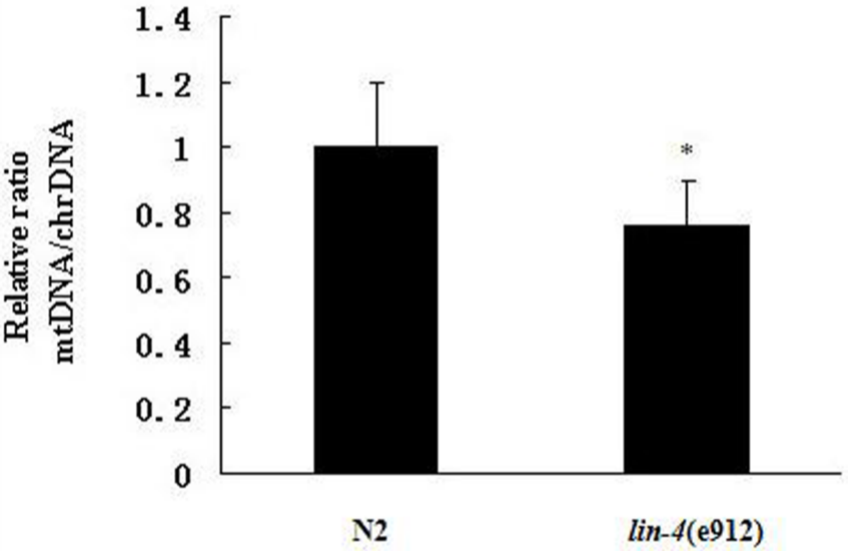
Effects of *lin-4* mutation on copy number of mtDNA. The mtDNA copy number was measured in wild-type (N2) and *lin-4* mutant (e912) worms. Values represent the mtDNA copy number per worm. At least three replicates were performed. * *p* < 0.05.

DCF assay experiments revealed that worms with a loss-of-function mutation in *lin-4* showed ROS accumulation (Figure 3). We examined the mtDNA copy number in *lin-4* mutants and wild-type animals by performing real-time PCR. The mtDNA copy number in *lin-4* mutants was significantly lower than that in the wild-type control (Figure 4).

### 2.3. Effect of *lin-4* on the Life Span and Locomotion of *C. elegans*

**Figure 5.**
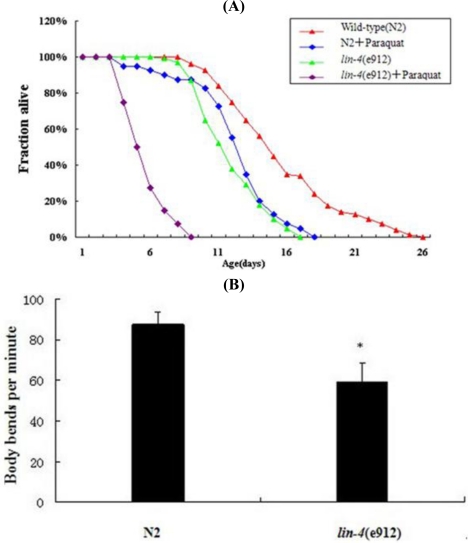
Effects of *lin-4* mutation on life span and locomotion. (**A**) Red, survival of wild-type (N2); blue, survival after addition of paraquat to culture dishes of N2; green, survival of *lin-4* mutants (e912); purple, survival after addition of paraquat to culture dishes of *lin-4* mutants (e912). N2: *n* = 95, *m* = 16.71, *M* = 25. *lin-4* (e912): *n* = 100, *m* = 9.42, *M* = 16. N2 + Paraquat: *n* = 115, *m* = 9.07, *M* = 15. *lin-4* (e912) + Paraquat: *n* = 112, *m* = 4.65, *M* = 8. (*n*: number; *m*: mean life; *M*: maximum life); (**B**) N2 and *lin-4* (e912) showed a mean of 85 and 58 body bends, respectively, over 1 minute. * *p* < 0.05.

As seen in Figure 5A, the mean and maximum life span of wild-type worms grown on NGM plates containing paraquat were, respectively, 7.64 d (*p* < 0.01) and 10 d (*p* < 0.01) lower than the corresponding values for wild-type worms grown on NGM plates. The mean and maximum life span of *lin-4* mutants grown on NGM plates containing paraquat were, respectively, 4.77 d (*p* < 0.01) and 8 d (*p* < 0.01) lower than the corresponding values for *lin-4* mutants grown on NGM plates. The mean and maximum life span of worms with a loss-of-function mutation in *lin-4* were, respectively, 7.29 d (*p* < 0.01) and 9 d (*p* < 0.01) lower than the corresponding values for the wild-type worms. Further, the mean and maximum life span of *lin-4* mutants grown on NGM plates containing paraquat were, respectively, 4.42 d (*p* < 0.01) and 7 d (*p* < 0.01) lower than the corresponding values for the wild-type worms grown on NGM plates containing paraquat. The mean and maximum life span of wild-type worms grown on NGM plates containing paraquat were, respectively, 0.35 d (*p* > 0.05) and 1 d (*p* > 0.05) lower than the corresponding values for *lin-4* mutants grown on NGM plates. The mean and maximum life span of *lin-4* mutants grown on NGM plates containing paraquat were, respectively, 12.06 d (*p* < 0.01) and 17 d (*p* < 0.01) lower than the corresponding values for wild-type worms grown on NGM plates. Additionally, assessment of the body-bend behavior of *C. elegans* showed that a loss-of-function mutation in *lin-4* caused severe movement defects (Figure 5B).

### 2.4. Discussion

In the present study, we showed that *lin-4* mutants had a remarkably reduced fat content and that this phenomenon also occurred when glucose or fatty acid was used as an energy source. SBP-1 is a homolog of the mammalian transcription factor Sterol response element binding protein (SREBP). SREBP is a key transcriptional regulator in the fat and sterol synthesis pathways [14]. Previous studies have shown that loss-of-function mutations in *C. elegans* SREBP are possibly correlated with decreased fat storage [10,15]. *O*-linked *N*-acetylglucosamine (*O*-GlcNAc) is thought of as a dynamic nuclear and cytosolic modulator of transcriptional and signal transduction events [16]. OGA is a key enzyme regulating *O*-GlcNAc cycling and is highly conserved in eukaryotic evolution from *Drosophila melanogaster* and *C. elegans* to rodents and man [13]. A recent study suggested that active cycling of *O*-GlcNAc by OGA-1 was required to maintain normal fat reserves in worms. In contrast, reduced fat accumulation was seen in mutant strains [13]. We speculate that the remarkable reduction in fat accumulation in *lin-4* mutants is due to the reduction in SBP-1 and OGA-1 mRNA levels.

Many studies have addressed the effect of *lin-4* on the life span of *C. elegans*, while none has reported the relationship between ROS and *lin-4*. We assayed *lin-4* mutants and wild-type worms for intracellular ROS and mtDNA copy number and found that mutations in *lin-4* resulted in ROS accumulation and a decreased mtDNA copy number. Worms with a loss-of-function mutation in *lin-4* had a life span that was significantly shorter than that of wild-type worms. Paraquat is a bipyridyl herbicide that consumes oxygen and generates superoxides [17]. We added paraquat to culture plates and observed a considerable shortening in life span, especially in *lin-4* mutants. This result shows that *lin-4* is required to prevent premature death. Previous studies have suggested that ROS possibly influences and controls the life span of animals. For example, *daf-2* and *isp-1* mutants live longer because of their low levels of ROS [18]. Similarly, *clk-1* mutants have low levels of accumulated byproducts from oxidative damage and extended life spans because of decreased ROS levels [19]. Mitochondria are major sources of ROS, and the mitochondria themselves can be damaged by ROS [30]. However, there is only indirect evidence linking genes encoded by mtDNA function with aging. This evidence is in the form of a series of mutations in nuclear genes that is sufficient to prove mtDNA involvement in the regulation of aging [21]. Therefore, we aimed to determine whether ROS and mtDNA influence the life span of *lin-4* mutants.

Locomotion of *C. elegans* is controlled by a subset of its nervous system, and manipulations at the genetic or neuronal level allow an insight into the inner workings of this control [22]. Previous studies revealed that physiological levels of oxidative stress are associated with a balance between beneficial and harmful effects, and that normal levels of oxidative stress in *C. elegans* may be optimized for locomotor activity [23,24]. In the mature central nervous system, oxidative stress, calcium influx, and glutamate excitotoxicity can induce apoptosis. High ROS levels are capable of damaging cellular components, proteins, and nucleic acids and can even cause necrosis [25]. These facts imply that a loss-of-function mutation in *lin-4* caused movement defects because of severe ROS accumulation.

## 3. Experimental Methods

### 3.1. Worms and Culture

Wild-type *C. elegans* Bristol (N2) and *lin-4* (e912) variant worms used in this study were obtained from the Caenorhabditis Genetics Center (Minnesota, USA). The *lin-4* (e912) variant is a *lin*-4 null mutant. Worms were grown on NGM (Nematode Growth Medium) agar plated with *E. coli* OP50 at 20 °C.

### 3.2. Nile Red Staining

Nematodes were bred on media with 50 ng/mL Nile red (MP Biomedicals, CA, USA) and *E. coli*. After 72 h of culture, worms were collected, treated with 0.2% paraformaldehyde (PFA) solution, and observed under a fluorescence microscope (DMRXA; Leica) with N3 filter [27,28]. Images of Nile red staining were acquired using a Nikon camera under identical settings and exposure times to allow direct comparisons. The relative fluorescence of the whole nematode body was determined densitometrically using Image-Pro^®^ Plus version 6.0, a commercially available software package (Media Cybernetics, Bethesda, MD, USA).

### 3.3. Effect of Fatty Acid

Worms were cultured on an NGM plate containing 1 mM fatty acid (stearic acid, oleic acid, or linoleic acid; WAKO, JAPAN) and 0.1% tergitol type NP-40 (Sigma, St. Louis, MO, USA) [12]. When the worms reached the young adult stage, they were subjected to Nile red analysis.

### 3.4. Effect of Glucose

Worms were cultured on an NGM plate with 5 mM glucose, and were continually fed increasing doses of glucose until they reached the young adult stage [12,29]. Young adult *C. elegans* were subjected to Nile red analysis.

### 3.5. Effect of Paraquat

Worms were cultured on an NGM plate containing 2 mM paraquat (Sigma) [30] and continued to grow into young adulthood with the dose. Young adult *C. elegans* were subjected to life span analysis.

### 3.6. Measurement of Intracellular ROS in *C. elegans*

Measurement of intracellular ROS in *C. elegans* was performed as previously described [31]. The amount of ROS was quantified using H2-DCF-DA (Sigma) as the molecular probe. H2-DCF-DA enters the cell, converts to H2-DCF, and is then rapidly oxidized by intracellular ROS to yield the fluorescent dye DCF. *C. elegans* worms were transferred to 2 mL of M9 buffer (22 mmol/L KH_2_PO_4_, 22 mmol/L Na_2_HPO_4_, 85 mmol/L NaCl, and 1 mmol/L MgSO_4_) that contained 10 μM CM-H2DCFDA and incubated for 30 min at 20 °C. To determine the fluorescence of DCF, fixed nematode samples were analyzed using a fluorescence microscope (excitation at 488 nm and emission at 510 nm). The relative fluorescence of the whole body was determined densitometrically using Image-Pro^®^ Plus version 6.0, a commercially available software package (Media Cybernetics).

### 3.7. Real-Time PCR of mtDNA

The mtDNA copy number was determined by quantitative PCR assay as described previously [32]. The primer sets used to amplify mtDNA and chrDNA were described previously [33]. Sequences and TaqMan probes are shown in Table 1. DNA was prepared using a DNA extraction kit (Promega, Madison, WI, USA), and the samples were analyzed on a 7300 Real-Time PCR System (Applied Biosystems, Foster City, CA USA). All values were calculated using the absolute quantification method. The mtDNA copy number was normalized to the number of nuclear DNA copies.

### 3.8. Life Span and Locomotion Assay

Life span assays were performed as described previously [34]. All life span analyses were conducted at 20 °C, starting from the L4 stage to young adult-stage worms. Death was defined as failure to move after being prodded with a platinum wire. For analysis of locomotion, worms were transferred to an agar plate without a bacterial lawn. Locomotion rate was quantified by counting the number of body bends produced by the worms in 1 min. Body bends were counted by observing flexing in the middle of the worm body.

### 3.9. Quantitative RT-PCR

RNA extraction, purification, and reverse transcription were performed for each sample as described [35]. Quantitative RT-PCR using the universal TaqMan probe was performed, and the results were analyzed using a 7300 Real-Time PCR System (Applied Biosystems) under the following conditions: Samples were incubated at 95 °C for 10 min for initial denaturation, followed by 40 cycles of amplification that were performed at 95 °C for 15 s and at 60 °C for 1 min. All data were calculated using standard relative quantification ΔΔCT methods [36], and actin was used as a control for normalization. All primers and probes for quantitative RT-PCR are listed in Table 2.

### 3.10. Statistical Analysis

All data are shown as mean ± SEM. Statistical analysis was performed using one-way ANOVA with the SPSS 12.0 statistical software package (SPSS Inc., USA). The level of significance was defined as *p* < 0.05.

## 4. Conclusion

In this study, we found that a loss-of-function mutation in *lin-4* in *C. elegans* led to a reduction in fat storage and decreased locomotion, and our findings also suggested that ROS may play an important role in the life span of *lin-4* mutants.

## Figures and Tables

**Table 1.** Sequences and TaqMan probes of mtDNA used in this study.

**Symbol**	**Wormbase**	**Primers**
GDP2 (nuclear)	K10B3.8.1	Probe:ACATAGTCGGCTCCAGAGGCTCC
		Sense: CGCAGCACAAGATCAAGGTAG
		Anti: AAGACTCCGGTGGACTCAAC
CYTB (mitochondria)	Mitochondrial	Probe:GGGTCAGCTTCAATAAACATCTCTGC
		Sense: GGTTATATTGCCACGGTGATTATG
		Anti:AATATCACTCTGGAACAATATGAACTG

**Table 2.** Sequences and target positions of the primers used for quantitative RT-PCR in this study.

**Symbol**	**Wormbase**	**Primers**
*Sbp-1*	Y47D3B.7	Probe:CTTCGCCGCCTTCTCCTTACTCCTCG
		Sense:CCACCACCTCATCACCACCAT
*Oga-1*	T20B5.3a	Anti: CCTTCCGCTGTCTCCTGAATCA
		Probe:CAGCCGAGTATTCACAGCCTATGGA
actin	T04C12.5	Sense:GAGCCTGTGCCTTCTGAGTTA
		Anti: CAGTGATAGTCTTTGATTTCTTATTTCCT
		Probe:CATCCTCCGTCTTGACTTGGCTGG
		Sense:CAATCTACGAAGGATATGCCCTC
		Anti: CTCAGCGGTGGTGGTGAA

## References

[1] Bartel DP (2004). MicroRNAs: genomics, biogenesis, mechanism, and function. Cell.

[2] Ambros V (2004). The functions of animal microRNAs. Nature.

[3] Wienholds E, Plasterk RH (2005). MicroRNA function in animal development. FEBS Lett.

[4] Heneghan HM, Miller N, Kerin MJ (2010). Role of microRNAs in obesity and the metabolic syndrome. Obes. Rev.

[5] Vella MC, Slack FJC (2005). Elegans microRNAs. Worm Book.

[6] Olsen PH, Ambros V (1999). The *lin-4* regulatory RNA controls developmental timing in Caenorhabditis elegans by blocking LIN-14 protein synthesis after the initiation of translation. Dev. Biol.

[7] Boehm M, Slack F (2005). A developmental timing microRNA and its target regulate life span in *C. elegans*. Science.

[8] Singhal V, Schwenk WF, Kumar S (2007). Evaluation and management of childhood and adolescent obesity. Mayo Clin. Proc.

[9] Speakman J, Hambly C, Mitchell S, Krol E (2008). The contribution of animal models to the study of obesity. Lab. Anim.

[10] McKay RM, McKay JP, Avery L, Graff JM (2003). *C elegans*: A model for exploring the genetics of fat storage. Dev. Cell.

[11] Brooks KK, Liang B, Watts JL (2009). The influence of bacterial diet on fat storage in *C. elegans*. PLoS One.

[12] Nomura T, Horikawa M, Shimamura S, Hashimoto T, Sakamoto K (2009). Fat accumulation in *Caenorhabditis elegans* is mediated by SREBP homolog SBP-1. Genes Nutr.

[13] Forsythe ME, Love DC, Lazarus BD, Kim EJ, Prinz WA, Ashwell G, Krause MW, Hanover JA (2006). *Caenorhabditis elegans* ortholog of a diabetes susceptibility locus: *oga-1* (O-GlcNAcase) knockout impacts O-GlcNAc cycling, metabolism, and dauer. Proc. Natl. Acad. Sci. USA.

[14] Eberlé D, Hegarty B, Bossard P, Ferré P, Foufelle F (2004). SREBP transcription factors: Master regulators of lipid homeostasis. Biochimie.

[15] Yang F, Vought BW, Satterlee JS, Walker AK, Jim Sun ZY, Watts JL, DeBeaumont R, Saito RM, Hyberts SG, Yang S, Macol C, lyer L, Tjian R, van den Heuvel S, Hart AC, Wagner G, Naar AM (2006). An ARC/Mediator subunit required for SREBP control of cholesterol and lipid homeostasis. Nature.

[16] Wells L, Whelan SA, Hart GW (2003). O-GlcNAc: A regulatory post-translational modification. Biochem. Biophys. Res. Commun.

[17] González-Polo RA, Rodríguez-Martín A, Morán JM, Niso M, Soler G, Fuentes JM (2004). Paraquat-induced apoptotic cell death in cerebellar granule cells. Brain Res.

[18] Hekimi S, Guarente L (2003). Genetics and the specificity of the aging process. Science.

[19] Braeckman BP, Houthoofd K, Brys K, Lenaerts I, de Vreese A, van Eygen S, Raes H, Vanfleteren JR (2003). No reduction of energy metabolism in Clk mutants. Mech. Ageing Dev.

[20] Ide T, Tsutsui H, Hayashidani S, Kang D, Suematsu N, Nakamura K, Utsumi H, Hamasaki N, Takeshita A (2001). Mitochondrial DNA damage and dysfunction associated with oxidative stress in failing hearts after myocardial infarction. Circ. Res.

[21] Beckman KB, Ames BN (1998). The free radical theory of aging matures. Physiol. Rev.

[22] Boyle JH, Cohen N (2008). *Caenorhabditis elegans* body wall muscles are simple actuators. Biosyststems.

[23] Finkel T, Holbrook NJ (2000). Oxidants, oxidative stress and the biology of aging. Nature.

[24] Murakami S, Murakami H (2005). The effects of aging and oxidative stress on learning behavior in *C. elegans*. Neurobiol. Aging.

[25] Kam PCA, Ferch NI (2000). Apoptosis: Mechanisms and clinical implications. Anaesthesia.

[26] Libina N, Berman JR, Kenyon C (2006). Tissue-specific activities of *C. elegans* DAF-16 in the regulation of lifespan. Cell.

[27] Hashimoto T, Horikawa M, Nomura T, Sakamoto K (2010). Nicotinamide adenine dinucleotide extends the lifespan of *Caenorhabditis elegans* mediated by *sir-2.1* and *daf-16*. Biogerontology.

[28] Horikawa M, Nomura T, Hashimoto T, Sakamoto K (2008). Elongation and desaturation of fatty acids are critical in growth, lipid metabolism, and ontogeny of *Caenorhabditis elegans*. J. Biochem.

[29] Lee SJ, Murphy CT, Kenyon C (2009). Glucose shortens the life span of *C. elegans* by downregulating DAF-16/FOXO activity and aquaporin gene expression. Cell Metab.

[30] Keaney M, Matthijssens F, Sharpe M, Vanfleteren J, Gems D (2004). Superoxide dismutase mimetics elevate superoxide dismutase activity *in vivo* but do not retard aging in the nematode *Caenorhabditis elegans*. Free Radic. Biol. Med.

[31] Kampkötter A, Nkwonkam CG, Zurawski RF, Timpel C, Chovolou Y, Watjen W, Kahl R (2007). Investigations of protective effects of the flavonoids quercetin and rutin on stress resistance in the model organism *Caenorhabditis elegans*. Toxicology.

[32] Miller KG, Alfonso A, Nguyen M, Crowell JA, Johnson CD, Rand JB (1996). A genetic selection for *Caenorhabditis elegans* synaptic transmission mutants. Proc. Natl. Acad. Sci. USA.

[33] Ye K, Ji CB, Lu XW, Ni YH, Gao CL, Chen XH, Zhao YP, Gu GX, Guo XR (2010). Resveratrol attenuates radiation damage in *Caenorhabditis elegans* by preventing oxidative stress. J. Radiat. Res.

[34] Dillin A, Crawford DK, Kenyon C (2002). Timing requirements for insulin/IGF-1 signaling in *C. elegans*. Science.

[35] Taubert S, van Gilst MR, Hansen M, Yamamoto KR (2006). A Mediator subunit, MDT-15, integrates regulation of fatty acid metabolism by NHR-49-dependent and independent pathways in *C. elegans*. Genes Dev.

[36] Livak KJ, Schmittgen TD (2001). Analysis of relative gene expression data using real-time quantitative PCR and the 2(-Delta Delta C(T)) method. Methods.

